# Adherence and virologic suppression during the first 24 weeks on antiretroviral therapy among women in Johannesburg, South Africa - a prospective cohort study

**DOI:** 10.1186/1471-2458-11-88

**Published:** 2011-02-08

**Authors:** Ziad El-Khatib, Anna Mia Ekstrom, Ashraf Coovadia, Elaine J Abrams, Max Petzold, David Katzenstein, Lynn Morris, Louise Kuhn

**Affiliations:** 1 Division of Global Health (IHCAR), Karolinska Institutet, Stockholm, Sweden; 2 AIDS Virus Research Unit, National Institute for Communicable Diseases (NICD), Johannesburg, South Africa; 3 Empilweni Services and Research Unit, Rahima Moosa Mother and Child Hospital, Faculty of Health Sciences, University of the Witwatersrand, Johannesburg, South Africa; 4 International Center for AIDS Programs, Mailman School of Public Health, Columbia University, New York, USA; 5 Nordic School of Public Health (NHV), Gothenburg, Sweden; 6 Division of Infectious Diseases, Stanford University, California, USA; 7 Gertrude H. Sergievsky Center, College of Physicians and Surgeons, and Department of Epidemiology, Mailman School of Public Health, Columbia University, New York, NY, USA

## Abstract

**Background:**

Adherence is a necessary part of successful antiretroviral treatment (ART). We assessed risk factors for incomplete adherence among a cohort of HIV-infected women initiating ART and examined associations between adherence and virologic response to ART.

**Methods:**

A secondary data analysis was conducted on a cohort of 154 women initiating non-nucleoside reverse transcriptase inhibitor (NNRTI)-based ART at a single site in Johannesburg, South Africa. Ninety women had been enrolled in a prevention of mother-to-child transmission (pMTCT) program and were exposed to single-dose nevirapine (sdNVP) >18 months earlier. Women were interviewed pre-treatment and clinical, virologic and adherence data were collected during follow-up to 24 weeks. Incomplete adherence to ART was defined as returning >5% of medications, estimated by pill counts at scheduled visits. Multivariable logistic regression analysis and unadjusted odds ratio (95%CI) were performed, using STATA/SE (ver 10.1).

**Results:**

About half of the women (53%) were <30 years of age, 63% had <11 years of schooling, 69% were unemployed and 37% lived in a shack. Seven percent of women had a viral load >400 copies/ml at 24 weeks and 37% had incomplete adherence at one or more visits. Incomplete adherence was associated with less education (p = 0.01) and lack of financial support from a partner (p = 0.02) after adjustment for confounders. Only when adherence levels dropped below 80% was there a significant association with viremia in the group overall (p = 0.02) although adherence <95% was associated with viremia in the sdNVP-exposed group (p = 0.03). The main reasons for incomplete adherence were being away from home, busy with other things and forgetting to take their medication.

**Conclusion:**

Virologic response to NNRTI-treatment in the cohort was excellent. However, women who received sdNVP were at greater risk of virologic failure when adherence was <95%. Women exposed to sdNVP, and those with less education and less social support may benefit from additional adherence counseling to ensure the long-term success of ART. More than 80% adherence may be sufficient to maintain virologic suppression on NNRTI-based regimens in the short-term, however complete adherence should be encouraged.

## Background

National and international organizations have committed to support antiretroviral treatment (ART) programs in sub-Saharan Africa (SSA)[1,2]. More than 60% of HIV-infected adults in SSA are women, who are disproportionately affected by the HIV-1 epidemic for both biological and socio-cultural reasons[1,3,4]. Although women are more likely to seek healthcare and initiate ART earlier than men,[4,5] they may be more likely to show incomplete adherence and discontinue ART during the first year on therapy[6]. Women's adherence to ART may be compromised by child-care responsibilities and dependency ratios,[7,8] economic pressures and lack of partner support[8,9]. Incomplete adherence to ART increases women's risk of virologic failure and subsequent clinical progression.

The association between adherence and virologic outcome is complex[10]. Earlier, unboosted protease inhibitor-based ART regimens required more than 95% adherence to ensure virologic suppression[11]. With today's non-nucleoside reverse transcriptase inhibitor (NNRTI)- and boosted protease inhibitor-based regimens a moderate adherence level (70-90%) may be adequate to achieve virologic suppression[12-14]. Bangsberg and co-workers[15] found, using a medical electronic assessment (MEMS), that >75% adherence may be sufficient to achieve virologic suppression among men receiving an NNRTI-based regimen in San Francisco. However, for optimal outcomes and to avoid disease progression[16-19], maximum level of adherence (100%) should always be the patient's goal and the provider's recommendations[12-14]. An additional factor for women that may jeopardize ART success is prior receipt of single-dose nevirapine (sdNVP) for prevention of mother-to-child transmission which can select drug resistance and jeopardize the efficacy of ART[20-23]. Little is known about the association between adherence level and virologic suppression among women exposed to sdNVP.

In this study we examined socio-economic characteristics associated with incomplete adherence, assessed through pharmacy pill counts, and HIV RNA quantified in plasma among women in a research cohort, from ART initiation through 24 weeks of treatment in Johannesburg, South Africa. The aim was to identify characteristics of women at higher risk of incomplete adherence to better target interventions to enhance adherence and sustain long-term ART success.

## Methods

### Setting and population

HIV-infected women were recruited into an earlier study in Johannesburg, South Africa, between July 2004 and May 2006, to assess whether previous sdNVP impacted the efficacy of ART[23]. Women who had received sdNVP as prophylaxis in a prior pregnancy 18 to 36 months earlier and women who had a pregnancy in the same interval but who did not receive sdNVP were eligible for enrollment. Other inclusion criteria included ART-naïve and (i) presenting with CD4+ cell counts <200 cells/mm^3^, (ii) CD4+ cell counts <350 cells/mm^3 ^and World Health Organization (WHO) stage III, or (iii) WHO stage IV disease. Exclusion criteria were (i) acute hepatitis, (ii) elevated liver function test values or (iii) a history of nevirapine toxicity[23]. Women were initiated onto therapy with nevirapine, stavudine and lamivudine unless co-treatment for tuberculosis was required in which case efavirenz was substituted for nevirapine. The primary results of this study have been previously reported[23]. All women provided written informed consent and the study was approved by the Institutional Review Boards of Columbia University, USA and the University of the Witwatersrand, South Africa.

### Study procedures

A baseline questionnaire was administered at enrolment prior to treatment initiation including questions on education level, socio-economic indicators, household members, previous pregnancies, previous live births and number of living children, marital status, quality of life (QOL)[24-26] and exposure to sdNVP. HIV RNA viral load (VL) and CD4+ cell counts were measured pre-treatment and WHO clinical stage was determined. Women attended the clinic monthly to 24 weeks and at each visit VL was measured and an adherence assessment was done by the study pharmacist. At week 24, another questionnaire was administered monitoring life events and QOL since ART initiation. CD4+ cell counts and VL ≤400 or >400 copies/ml were the primary treatment outcomes.

### Adherence and virologic failure assessment

Pill counts[27] were performed by the study pharmacist, who counted the number of remaining pills for each drug separately at each drug refill visit. Refills were scheduled at weeks 2, 4, 8, 12, 16, 20 and 24 from the start of treatment. Pill count-based adherence was assessed by calculating the average combined pill count for the three drugs at each visit, using the formula [Adherence = (Number of pills dispensed - Number of pills returned) × 100)/(Number of pills prescribed daily × Number of days between pharmacy visits)].

In our study, the adherence level of women was assessed at the seven time points. Patients were defined as incompletely adherent, if they ever had an average pill count of returning back >5% of their prescribed pills to the pharmacy, indicating an adherence level of <95% adherence at any visit. We also performed additional analyses of levels of adherence indicated by the percentage of returned pills from 0%, to >5%, >10%, >20% and >30% on the outcome of VL >400 copies/ml at week 24. This analysis was done first among all women and then among women exposed vs. not exposed to sdNVP. Patients were asked a battery of questions to assess QOL, where the sum of questions varied from 12 (very good QOL) to 55 (the poorest level of QOL). These responses were categorized as 1) 12-23 (good), 2) 24-35 (intermediate) or 3) 36-55 (poor) to analyze the relationship between QOL, adherence and virologic response.

Viral load was measured using the Roche Amplicor assay (Roche Diagnostics, version 1.5) using the standard assay (lower limit of detection, 400 copies/ml) before commencement of treatment and the ultrasensitive assay (lower limit of detection, 50 copies/ml) after commencement of treatment. CD4+ cell count was measured by flow cytometry[23]. Viral load was assessed pre-ART initiation and every four weeks until week 24 and VL >400 copies/ml was defined as virologic failure. CD4+ cell count was assessed pre-ART initiation and every 12 weeks[23].

### Adherence support

Special attention was given to adherence counseling. A patient contract was drawn up in which the participant agreed with the clinical staff to adhere as closely as possible to medication guidelines. A booklet including HIV and treatment information considered necessary for adequate adherence was developed and participants were evaluated for their understanding of this information prior to starting therapy. Pill-boxes and diary cards designed to suit the regimen were given to all participants. Each post-treatment visit included a pharmacist's review of dosing and adherence, a pill-count of returned drugs, and all members of the clinical team offered support around adherence throughout the study[23].

### Data analysis

Characteristics of the participants (including demographics, socio-economic and pre-treatment clinical factors) were compiled. Associations between incomplete adherence, virologic failure and these other characteristics were assessed using unadjusted odds ratios (OR) with 95% confidence intervals (CI) and p-values. Backward-selection multivariable logistic regression analysis was performed including factors with p-values ≤0.10 and then repeated for those variables yielding p-values ≤0.05. Associations between incomplete adherence at different thresholds and virologic failure were assessed using unadjusted odds ratios with 95% CI. The statistical analysis was performed using STATA/SE (ver 10.1).

## Results

### Characteristics of women pre-ART initiation

A total of 154 women participated in this study. Of these, 147 (95%) had pill count assessment data for at least five out of seven visits during the study period (i.e. the first 24 weeks on ART); data on seven patients (5%) were missing for more than five visits and these were excluded from the final analysis. The socio-economic and clinical characteristics of these seven women, did not differ from the women included in the analysis (data not shown). Most of the 147 women were born in South Africa (88%), 53% were below 30 years of age, and 63% had less than 11 years of schooling. In terms of income, 69% were unemployed and one third (37%) lived in an informal dwelling (shack). Most (83%) women reported being single, 75% had been pregnant at least twice and 40% had at least two living children. When we asked about financial support from a partner or a husband, less than half of single women (38%) and a majority of married women (84%) reported receipt of support. The majority had more than four of the pre-specified household assets: indoor tap water (50%), toilet (42%), electricity (84%), a refrigerator (66%), radio (82%), TV (82%) or a landline telephone (11%), defining higher socio-economic status. Seventy percent had disclosed their HIV status to someone at home and 85% had a treatment buddy. Ninety women had been exposed to sdNVP in their previous pregnancy. The median levels of VL and CD4 cell count pre-ART initiation were 110,000 copies/ml (range 760 - >750,000) and 166 cells/μl (range 2 - 430) respectively. Less than a quarter (23%) scored poorly on QOL assessments at baseline.

### Risk factors for incomplete adherence

Fifty-five patients (37%) demonstrated incomplete adherence, as defined by ever returning >5% of their pills at any visit through 24 weeks. Univariable analysis examining pre-ART factors associated with incomplete adherence is shown in Table 1. In multivariable analysis, two factors remained significantly associated with incomplete adherence, namely, education level ≤grade 11 (OR = 2.6; 95% CI 1.2-5.6; p = 0.01) and not receiving financial support from a partner or a husband (OR = 2.3; 95% CI 1.1-4.8; p = 0.02). Women who had received sdNVP as prophylaxis in a prior pregnancy were less likely to demonstrate incomplete adherence (OR = 0.5; 95% CI 0.2-1.0; p = 0.05) but this association did not persist after adjustment for confounders.

**Table 1:** Pre-antiretroviral therapy (ART) characteristics and incomplete adherence over first 24 weeks of therapy (ever with pill count <95% past 24 weeks)

	Number (%) of those with complete adherence (n = 92)	Number (%) of those with incomplete adherence (n = 55)	Univariable odds ratio (95% CI); p-value
**Demographics Born in South Africa**			
No (18; 12%)	8 (9%)	10 (18%)	1
Yes (128; 88%)	83 (91%)	45 (82%)	0.4 (0.2-1.2); 0.10
**Age (years)**			
<30 (78; 53%)	50 (54%)	28 (51%)	1
≥30 (69; 47%)	42 (46%)	27 (49%)	1.2 (0.6-2.2); 0.69
**Education level**			
> grade 11 (54; 37%)	41 (45%)	13 (24%)	**1**
≤ grade 11 (91; 63%)	50 (55%)	41 (76%)	**2.6 (1.2-5.6); 0.01**
**Marital status**			
Single/divorced/widow (122; 83%)	74 (80%)	48 (87%)	1
Married/partner (25; 17%)	18 (20%)	7 (13%)	0.6 (0.2-1.5); 0.29
**Socio-economic status (SES) Employed**			
No (101; 69%)	64 (70%)	37 (67%)	1
Yes (46; 31%)	28 (30%)	18 (33%)	1.1 (0.5-2.3); 0.77
**Type of housing**			
House/flat/rented room (93; 63%)	63 (68%)	30 (55%)	1
Shack/outbuilding (54; 37%)	29 (32%)	25 (45%)	1.8 (0.9-3.6); 0.09
**Number of children living at home**			
0-1 child (89; 61%)	57 (62%)	32 (58%)	1
≥ 2 children (58; 39%)	35 (38%)	23 (42%)	1.2 (0.6-2.3); 0.65
**Lack of financial support from partner/husband**			
Support (67; 45%)	49 (53%)	18 (33%)	**1**
Lack of support (80; 55%)	43 (47%)	37 (67%)	**2.3 (1.1-4.8); 0.02**
**Water source inside home**			
No (74; 50%)	41 (45%)	33 (60%)	1
Yes (73; 50%)	51 (55%)	22 (40%)	0.5 (0.3-1.1); 0.07
**Toilet at home**			
No (86; 58%)	51 (55%)	35 (64%)	1
Yes (61; 42%)	41 (45%)	20 (36%)	0.7 (0.4-1.4); 0.33
**Electricity at home**			
No (23; 16%)	13 (14%)	10 (18%)	1
Yes (124; 84%)	79 (86%)	45 (82%)	0.7 (0.3-1.8); 0.51
**Refrigerator at home**			
No (50; 34%)	27 (29%)	23 (42%)	1
Yes (97; 66%)	65 (71%)	32 (58%)	0.6 (0.3-1.2); 0.12
**Radio at home**			
No (28; 19%)	15 (16%)	13 (24%)	1
Yes (119; 81%)	77 (84%)	42 (76%)	0.6 (0.3-1.5); 0.28
**TV at home**			
No (27; 18%)	12 (13%)	15 (27%)	**1**
Yes (120; 82%)	80 (87%)	40 (73%)	**0.4 (0.2-1.0); 0.03**
**Landline telephone**			
No (131; 89%)	82 (89%)	49 (89%)	1
Yes (16; 11%)	10 (11%)	6 (11%)	1.0 (0.3-2.9); 0.99
**Pre-treatment clinical characteristics Exposed to sdNVP**			
No (57; 39%)	30 (33%)	27 (49%)	**1**
Yes (90; 61%)	62 (67%)	28 (51%)	**0.5 (0.2-1.0); 0.05**
**VL level, pre-ART (copies/ml)**	127,500	97,000	
Median (range)	(760 - >750,000)	(6,600 - >750,000)	p = 0.17
**CD4 level (cells/μl)**			
Median (range)	155 (7 - 430)	189 (2 - 324)	p = 0.16

**Felt too sick to go to work or to do the daily activities, during the last 30 days**			
No (104; 71%)	64 (70%)	40 (73%)	1
Yes (43; 29%)	28 (30%)	15 (27%)	0.9 (0.4-1.8); 0.68*
**Traditional medicine/herbs in the last 3 months**			
No (126; 87%)	79 (86%)	47 (89%)	1
Yes (19; 13%)	13 (14%)	6 (11%)	0.8 (0.3-2.2); 0.63
**Quality of life**			
Good (score 12-23) (38; 26%)	27 (29%)	11 (20%)	1
Intermediate (score 24-35) (75; 51%)	43 (47%)	32 (58%)	1.8 (0.8-4.3); 0.16
Poor (score 36-55) (34; 23%)	22 (24%)	12 (22%)	1.3 (0.5-3.6); 0.57
**Disclosure and social support Disclosed HIV status**			
**No **(44; 30%)	27 (29%)	17 (31%)	1
Yes (102; 70%)	65 (71%)	37 (69%)	0.9 (0.4-1.9); 0.79
**Treatment buddy**			
No (22; 15%)	11 (12%)	11 (20%)	1
Yes (125; 85%)	81 (88%)	44 (80%)	0.5 (0.2-1.4); 0.19
**Another adult at home living with HIV**			
No (46; 57%)	29 (60%)	17 (51%)	1
Yes (35; 43%)	19 (40%)	16 (49%)	1.4 (0.5-3.9); 0.50*
**Another child at home living with HIV**			
No (12; 12%)	9 (15%)	3 (8%)	1
Yes (87; 88%)	53 (85%)	34 (92%)	1.9 (0.4-11.8); 0.53*

TV, television, sdNVP, single dose nevirapine; VL, viral load; ART, antiretroviral therapy

* 2-sided Fisher's exact test

Characteristics reported at 24 weeks were also investigated as potential correlates of incomplete adherence (Table 2). The remaining risk factors associated with a trend towards incomplete adherence with p-values ranging from 0.06 to 0.10 were: birth outside South Africa, living in informal housing, not having a water source inside the home, low socio-economic status, and reporting a divorce or separation during first 24 weeks on ART.

**Table 2:** Characteristics at 24 weeks post-antiretroviral therapy (ART) initiation and incomplete adherence (ever with pill count <95% past 24 weeks)

Type of exposure	Number (%) in group who had complete adherence (n = 92)	Number (%) in group who had incomplete adherence (n = 55)	Univariable odds ratio (CI 95%) p value
**Quality of life**			
Good (score 12-23) (95; 65%)	64 (70%)	31 (56%)	1
Intermediate (score 24-35) (45; 31%)	25 (27%)	20 (36%)	1.6 (0.8-3.4); 0.18
Poor (score 36-55) (6; 4%)	2 (2%)	4 (8%)	4.1 (0.5-47.2); 0.18*
**Life events since ART initiation Moved to a new accommodation**			
No (113; 77%)	73 (80%)	40 (73%)	1
Yes (33; 23%)	18 (20%)	15 (27%)	1.5 (0.7-3.4); 0.30
**Lost job**			
No (138; 95%)	88 (97%)	50 (91%)	1
Yes (8; 5%)	3 (3%)	5 (9%)	2.9 (0.5-19.5); 0.15*
**Physically attacked**			
No (133; 91%)	83 (91%)	50 (91%)	1
Yes (13; 9%)	8 (8%)	5 (9%)	1.0 (0.3-3.8); 1.00*
**Treated badly because of HIV status**			
No (129; 88%)	79 (87%)	50 (91%)	1
Yes (17; 12%)	12 (13%)	5 (9%)	0.7 (0.2-2.2); 0.60*
**Got divorced/separated**			
No (118; 80%)	78 (85%)	40 (73%)	1
Yes (29; 20%)	14 (15%)	15 (27%)	2.1 (0.9-4.8); 0.08
**Death of a child since ART initiation**			
No (140; 96%)	88 (97%)	52 (95%)	1
Yes (6; 4%)	3 (3%)	3 (5%)	1.7 (0.2-13.1); 0.67*
**Death of a family member since ART initiation**			
No (114; 78%)	72 (79%)	42 (76%)	1
Yes (32; 22%)	19 (21%)	13 (24%)	1.2 (0.5-2.6); 0.70
**Cumulative number of life events**			
0 (63; 43%)	42 (46%)	21 (38%)	1
1 (52; 36%)	34 (37%)	18 (33%)	1.1 (0.5-2.3); 0.89
2 (15; 10%)	7 (8%)	8 (15%)	2.3 (0.7-7.3); 0.15
≥3 (16; 11%)	8 (9%)	8 (14%)	2.0 (0.6-6.2); 0.22

ART, antiretroviral therapy

* 2-sided Fisher's exact test

### Risk factors for viral load >400 copies/ml at week 24

Eleven patients (7%) had VL >400 copies/ml at 24 weeks, six of whom (55%) were considered to be incompletely adherent during the study period. As shown in Table 3 there were trends between the viral outcome and incomplete adherence during the first 24 weeks on therapy (OR = 2.1; 95% CI 0.5-9.3; p = 0.33), an education level below or equal to grade 11 (OR = 2.9; 95% CI 0.6-28.0; p = 0.21), living in an informal settlement (OR = 2.2; 95% CI 0.5-9.6; p = 0.21) and a death in the family during the first 24 weeks on ART (OR = 3.3; 95% CI 0.7-14.0; p = 0.06). As has been reported previously, there was no significant difference in viral suppression between women exposed to sdNVP 18-36 months earlier relative to exposed women with a pregnancy within the same interval[23].

**Table 3:** Associations between pre-ART characteristics and viral load >400 copies/ml at week 24

Type of exposure	Number (%) in women with VL≤400 copies/ml at week 24 (%)	Number (%) in women with VL > 400 copies/ml at week 24 (%)	Univariable odds ratio (CI 95%) p-value
**Pre-ART initiation (n;%)**			

**Demographics**			
**Ever reported pill count level <95% over 24 weeks**			
No (92; 63%)	87 (64%)	5 (45%)	1
Yes (55; 37%)	49 (36%)	6 (55%)	2.1 (0.5-9.3); 0.33*
**Born in South Africa**			
No (18; 12%)	15 (11%)	3 (27%)	1
Yes (128; 88%)	120 (89%)	8 (73%)	0.3 (0.1-2.2); 0.14*
**Age (years)**			
<30 (78; 53%)	70 (51%)	8 (72.7%)	1
≥30 (69; 47%)	66 (49%)	3 (27.3%)	0.4 (0.1-1.7); 0.22*
**Education level**			
> grade 11 (54; 37%)	52 (39%)	2 (18%)	1
≤ grade 11 (91; 63%)	82 (61%)	9 (82%)	2.9 (0.6-28.0); 0.21*
**Marital status**			
Single/divorced/widow (122; 83%)	114 (84%)	8 (73%)	1
Married/partner (25; 17%)	22 (16%)	3 (27%)	1.9 (0.3-8.9); 0.40*
**Socio-economic status (SES)** **Employed**			
No (101; 69%)	95 (70%)	6 (55%)	1
Yes (46; 31%)	41 (30%)	5 (45%)	1.9 (0.4-8.0); 0.32*
**Type of housing**			
House/flat/rented room (93; 64%)	88 (65%)	5 (45%)	1
Shack/outbuilding (54; 37%)	48 (35%)	6 (55%)	2.2 (0.5-9.6); 0.21*
**Number of children living at home**			
0-1 child (89; 60%)	80 (59%)	9 (82%)	1
≥ 2 children (58; 40%)	56 (41%)	2 (18%)	0.3 (0-1.6); 0.20*
**Lack of financial support from partner/husband**			
Support (67; 45%)	62 (45%)	5 (45%)	1
No support (80; 55%)	74 (54%)	6 (55%)	1.00 (0.2-4.1); 1.00*
**Water source inside home**			
No (74; 50%)	68 (50%)	6 (55%)	1
Yes (73; 50%)	68 (50%)	5 (45%)	0.8 (0.2-3.5); 1.00*
**Toilet at home**			
No (86; 59%)	78 (57%)	8 (73%)	1
Yes (61; 41%)	58 (43%)	3 (27%)	0.5 (0.1-2.2); 0.36*
**Electricity at home**			
No (23; 16%)	22 (16%)	1 (9%)	1
Yes (124; 84%)	114 (84%)	10 (91%)	1.9 (0.2-87.5); 1.0*
**Refrigerator at home**			
No (50; 34%)	47 (35%)	3 (27%)	1
Yes (97; 66%)	89 (65%)	8 (73%)	1.4 (0.3-8.6); 0.75*
**Radio at home**			
No (28; 19%)	26 (19%)	2 (18%)	1
Yes (119; 81%)	110 (81%)	9 (82%)	1.1 (0.2-10.7); 1.00*
**TV at home**			
No (27; 18%)	25 (18%)	2 (18%)	1
Yes (120; 82%)	111 (82%)	9 (82%)	1.0 (0.2-10.2); 1.00*
**Landline telephone**			
No (131; 89%)	121 (89%)	10 (91%)	1
Yes (16; 11%)	15 (11%)	1 (9%)	0.8 (0-6.5); 1.00*
**Pre-treatment clinical characteristics**			
**Exposed to sdNVP**			
No (57; 39%)	51 (38%)	6 (55%)	1
Yes (90; 61%)	85 (62%)	5 (45%)	0.5 (0.1-2.1); 0.34*
**VL level (copies/ml) pre-ART**			
Median (range)	110,000 (760 - >750,000)	92,300 (6,600 - 359,000)	p = 0.34
**CD4 cell count - pre-ART**			
Median (range)	164 (2 - 430)	191 (7 - 355)	p = 0.91

**Quality of life (pre-ART)**			
Good (score 12-23) (38; 26%)	34 (25%)	4 (36%)	1
Intermediate (score 24-35) (75; 51%)	70 (51%)	5 (46%)	0.6 (0.1-3.3); 0.48*
Poor (score 36-55) (34; 23%)	32 (24%)	2 (18%)	0.5 (0-4.0); 0.68*
**Disclosure and social support Disclosed HIV status**			
No (44; 30%)	42 (31%)	2 (20%)	1
Yes (102; 70%)	94 (69%)	8 (80%)	1.8 (0.3-17.9); 0.72*
**Treatment buddy**			
No (22; 15%)	21 (15%)	1 (9%)	1
Yes (125; 85%)	115 (85%)	10 (91%)	1.8 (0.2-93.0); 1.00*
**Another adult at home, living with HIV**			
No (46; 57%)	43 (57%)	3 (50%)	1
Yes (35; 43%)	32 (43%)	3 (50%)	1.3 (0.2-10.7); 1.00*
**Another child at home, living with HIV**			
No (12; 12%)	12 (13%)	0	1
Yes (87; 88%)	79 (87%)	8 (100%)	NA; 0.59*
**At week 24**			
**Quality of life (post-ART)**			
Good (score 12-23) (95; 65%)	88 (65%)	7 (64%)	1
Intermediate (score 24-35) (45; 31%)	42 (31%)	3 (27%)	0.9 (0.1-4.2); 1.00*
Poor (score 36-55) (6; 4%)	5 (4%)	1 (9%)	2.5 (0-27.4); 0.40*
**Moved to a new accommodation**			
No (113; 77%)	105 (78%)	8 (73%)	1
Yes (33; 23%)	30 (22%)	3 (27%)	1.3 (0.2-5.9); 0.71*
**Lost job**			
No (138; 95%)	128 (95%)	10 (91%)	1
Yes (8; 5%)	7 (5%)	1 (9%)	1.8 (0-16.7); 0.47*
**Physically attacked**			
No (133; 91%)	122 (90%)	11 (100%)	1
Yes (13; 9%)	13 (10%)	0	NA
**Treated badly because of HIV status**			
No (129; 88%)	120 (89%)	9 (82%)	1
Yes (17; 12%)	15 (11%)	2 (18%)	1.8 (0.2-9.8); 0.62*
**Got divorced/separated**			
No (118; 80%)	109 (80%)	9 (82%)	1
Yes (29; 20%)	27 (20%)	2 (18%)	0.9 (0.1-4.7); 1.00*
**A child died since ART initiation**			
No (140; 96%)	130 (96%)	10 (91%)	1
Yes (6; 4%)	5 (4%)	1 (9%)	2.6 (0.1-26.6); 0.38*
**Any family member died since ART initiation**			
No (114; 78%)	108 (80%)	6 (55%)	1
Yes (32; 22%)	27 (20%)	5 (45%)	3.3 (0.7-14.0); 0.06*
**Cumulative number of life events**			
0 (63; 43%)	59 (44%)	4 (36%)	1
1 (52; 36%)	48 (36%)	4 (36%)	1.2 (0.2-6.9); 1.00*
2 (15; 10%)	14 (10%)	1 (9%)	1.1 (0-11.8); 1.00*
≥3 (16; 11%)	14 (10%)	2 (19%)	2.1 (0.2-16.3); 0.59*

TV, television, sdNVP, single dose nevirapine; VL, viral load; ART, antiretroviral therapy

* 2-sided Fisher's exact test

### Different adherence levels and viral load >400 copies/ml at week 24

Correlations between VL >400 copies/ml at week 24 and adherence defined using varying thresholds are shown in Table 4a. In the group overall, there was a significant association between VL >400 copies/ml and ever having an adherence level <80% (p = 0.01) or <70% (p = 0.01). Next, we assessed the correlation between VL >400 copies/ml and adherence defined using varying thresholds stratifying women based on their exposure to sdNVP (Table 4b). For women who had received sdNVP, there was a significant association between VL >400 copies/ml at week 24 and adherence levels <95%, <90%, <80%, and <70% (p ≤ 0.03). For women who had not been exposed to sdNVP, there was no significant association at any threshold.

**Table 4:** Correlation between different adherence levels and VL >400 copies/ml at week 24: a) among all women and b) among women exposed vs. not exposed to sdNVP separately

a)	All women		
Percent adherence during any of the visits 2- 24 weeks	≤ 400 copies/ml	>400 copies/ml	Univariable odds ratio (CI 95%); p value
**100% (n = 28)**	27 (20%)	1 (9%)	1
**Ever <100% (n = 119)**	109 (80%)	10 (91%)	2.5 (0.3-111.4); 0.69*
**>95% (n = 92)**	87 (64%)	5 (45%)	1
**Ever <95% (n = 55)**	49 (36%)	6 (55%)	2.1 (0.5-9.3); 0.33*
**>90% (n = 105)**	99 (73%)	6 (55%)	1
**Ever <90% (n = 42)**	37 (27%)	5 (45%)	2.2 (0.5-9.3); 0.30*
**>80% (n = 122)**	116 (85%)	6 (55%)	**1**
**Ever <80% (n = 25)**	20 (15%)	5 (45%)	4.8 (1.0-20.8); 0.02*
**>70% (n = 137)**	129 (95%)	8 (73%)	**1**
**Ever <70% (n = 10)**	7 (5%)	3 (27%)	6.9 (1.0-37.7); 0.03*

**b)**	**Exposed to sdNVP**		
**Percent adherence during any of the visits 2- 24 weeks**	**≤ 400 copies/ml**	**> 400 copies/ml**	**Univariable odds ratio** **(CI 95%); p value**

**100% (n = 18)**	18 (21%)	0	1
**Ever <100% (n = 72)**	67 (79%)	5 (100%)	NA: 0.58*
**>95% (n = 62)**	61 (72%)	1 (20%)	**1**
**Ever <95% (n = 28)**	24 (28%)	4 (80%)	10.2 (0.9-508.5); 0.03*
**>90% (n = 69)**	68 (80%)	1 (20%)	**1**
**Ever <90% (n = 21)**	17 (20%)	4 (80%)	16 (1.4-798.9); 0.01*
**>80% (n = 75)**	74 (87%)	1 (20%)	**1**
**Ever <80% (n = 15)**	11 (13%)	4 (80%)	26.9 (2.2-1341.7); <0.01*
**>70% (n = 83)**	80 (94%)	3 (60%)	**1**
**Ever <70% (n = 7)**	5 (6%)	2 (40%)	10.7 (0.7-112.3); 0.01*

	**Not exposed to sdNVP**		
	**≤ 400 copies/ml**	**> 400 copies/ml**	**Univariable odds ratio** **(CI 95%); p value**

**100% (n = 10)**	9 (18%)	1 (17%)	1
**Ever <100% (n = 47)**	42 (82%)	5 (83%)	1.1 (0.1-56.2); 1.00*
**>95% (n = 30)**	26 (51%)	4 (67%)	1
**Ever <95% (n = 27)**	25 (49%)	2 (33%)	0.5 (0-4.1); 0.67*
**>90% (n = 36)**	31 (61%)	5 (83%)	1
**Ever <90% (n = 21)**	20 (39%)	1 (17%)	0.3 (0-3.1); 0.40*
**>80% (n = 47)**	42 (82%)	5 (83%)	1
**Ever <80% (n = 10)**	9 (18%)	1 (17%)	0.9 (0-10.0); 1.00*
**>70% (n = 54)**	49 (96%)	5 (83%)	1
**Ever <70% (n = 3)**	2 (4%)	1 (17%)	4.9 (0.1-106.0); 0.29*

sdNVP, single dose nevirapine; VL, viral load

* 2-sided Fisher's exact test

### Barriers to adherence

**Figure 1:**
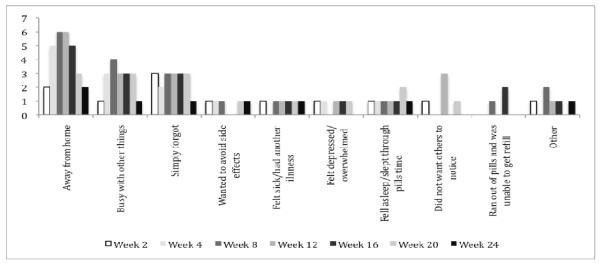
**Percentage of the most frequent reasons for missing pills over weeks 2-24**.

Potential barriers to adherence endorsed by study participants are shown in Figure 1. The women reported three main reasons for missing their medication: being away from home, being busy with other things and simply forgetting. The most common reason for missing pills, *viz *being away from home, varied in importance over the first 24 weeks. Other reasons for missing daily medications remained similar over weeks 2-20 diminishing in importance by week 24.

## Discussion

In this cohort of women starting ART in Johannesburg and who received extensive adherence support, we explored the association between socio-economic characteristics, adherence and virologic failure during the first 24 weeks of treatment. There was a high level of adherence despite the social and economic challenges faced by these participants, many of whom were single mothers living in disadvantaged economic circumstances. These observations are consistent with other studies from SSA [28-31] and reflect patients' commitment to HIV care in South Africa[28].

Reduced adherence, measured as a percent of doses missed in a given time period, has been associated with virologic failure. The relationship between adherence and virologic failure appears to be affected by the classes of drugs used and may differ between early and later treatment[32,33]. In our data, it appeared that previous use of sdNVP may affect the relationship between adherence and virologic failure of an NNRTI-containing regimen. In the total study population, less than 80% adherence was significantly associated with virologic failure. However, when women were stratified by exposure to sdNVP, the effect of adherence on virologic failure was strongest among the sdNVP exposed women, where anything less than 95% adherence was significantly associated with increased virologic failure. This might be due to the selection and persistence of NVP resistance mutations, albeit as minority species undetected in consensus sequence[34]. In addition, the higher the level of adherence to NNRTI-based therapy, the lower the risk of drug resistance and virologic failure[18,32]. This contrasts with the unexposed women among whom there was no association between reduced levels of adherence and virologic failure. This difference may be a result of the relatively small number of unexposed women (n = 57) and should be confirmed in larger prospective studies. Nevertheless, our findings raise the question of whether drug resistance selected by sdNVP may be partially overcome with complete adherence.

The primary study was designed to assess the effect of sdNVP on virologic response to therapy. Although exposure to sdNVP in the prior 18-36 months was not associated with a reduced likelihood of achieving and sustaining viral suppression, women with minority K103N mutations before treatment had a reduced durability of virologic suppression[23] as observed in other studies[20,22,35,36]. Some studies, however, have reported poor virologic response to NNRTI-based therapy even if exposure is quite distant[37]. These differences between studies may, in part be explained by different levels of adherence. If the effects of incomplete adherence on virologic suppression depend on past drug exposure, as suggested by our data, studies with less attention to sustaining high levels of adherence may detect decreased efficacy of ART among those with past exposure to sdNVP. Since current guidelines recommend that pregnant women with <350 CD4+ cells/mm^3 ^receive ART, rather than sdNVP, there is less basis to be concerned about the impact of sdNVP on later treatment outcomes[38]. Adherence level to ART is found to be high among HIV infected pregnant women[39] compared to non-pregnant women[40,41]. However, ART is a life-time treatment and sustaining high adherence level should remain the ultimate goal.

The women who had participated in the pMTCT program were more likely to be adherent to ART in univariable analysis although this association did not remain after adjustment for social characteristics. Increased treatment preparation in pMTCT programs may provide women with information about ART and adherence as shown in a study in Uganda[42]. Another study[43] at a rural district hospital in South Africa demonstrated that women provided with partner support and complete pMTCT information were more likely to take sdNVP. Thus, programs that provide sdNVP for pMTCT and provide women with increased treatment education, information and support are likely to achieve better adherence on ART. Here, women, with less education were at higher risk for incomplete adherence. Other studies of women[44] and of men and women[45] found independent associations between education, health literacy and adherence to ART. An additional concern is women's understanding of their children's treatment needs, where poor education may pose an additional risk for inadequate administration of ART to their infected children[46].

In Johannesburg, less education, living in an informal setting and providing care for at least two children with uncertain partner support, were each associated with reduced adherence and an increased need for support[47]. Ware and co-workers[48] found, in a large ethnographic study in Nigeria, Tanzania and Uganda, that social support enhances adherence. Also Merenstein and co-workers[7] found that having the sole responsibility for a child reduces adherence among women in the United States. Lack of potable water at home as reported by Ellis and co-workers in Nairobi was associated with incomplete adherence among both adults and children[49]. In this study, we observed that patients with low socio-economic status, assessed by living in an informal dwelling, the absence of a TV or lack of potable water at home were each associated with incomplete adherence although these did not retain significance in the final model, possibly because of the small sample size. The three main reasons for missing pills were being away from home, being busy with other things and simply forgetting, which are consistent with the findings of a review by Mills and co-workers[50].

There are several limitations to the generalizability of this study. Postpartum women are a vulnerable population who may have particular difficulty in obtaining access to ART due to financial challenges as described by Rosen and co-workers[51]. Here we followed a relatively small number of women for only six months and did not monitor changes in socioeconomic status, relationships, employment or depression over time. Rather, we relied on baseline responses and recall at six months. Reimbursement for transport costs, estimated to be ~25% of the patients' cost of ART[51], may have improved adherence to treatment[52]. In addition, this was a clinical research study in which attention and resources were devoted to reinforcing adherence[53], so it is likely that incomplete adherence may be more prevalent in a routine HIV care setting. Further limitations include focus only on a single measure of adherence and uncertain validity of some of the co-factors investigated, including QOL. Adherence to ART is a dynamic process influenced by psychosocial and socio-economic factors that need further investigation[54].

## Conclusions

In conclusion, women receiving an NNRTI-based regimen may achieve virologic suppression with >80% adherence. However, higher levels of adherence may be necessary among women who have been exposed to sdNVP. Women starting on ART with less education and lower socio-economic level were also at a higher risk of incomplete adherence to ART over the first six months of treatment and special adherence counseling and interventions may be needed to ensure sufficient support for long-term sustainability of treatment response among these women.

## Competing interests

The authors declare that they have no competing interests.

## Authors' contributions

EJA, AC, LM and LK participated in study design, ethical clearance and data collection. ZEK, AME, MP, DK, LM and LK participated in data analysis. All authors read and approved the final manuscript.

## Pre-publication history

The pre-publication history for this paper can be accessed here:

http://www.biomedcentral.com/1471-2458/11/88/prepub
